# The diagnostic accuracy of touch imprint cytology for sentinel lymph node metastases of breast cancer: An up-to-date meta-analysis of 4,073 patients

**DOI:** 10.1515/med-2023-0827

**Published:** 2023-10-30

**Authors:** Qing-Qing Yang, Yun-Gang Hu, Chao-Hua Hu, Yun-Tao Han, Hao-Yuan Shen

**Affiliations:** Department of Thyroid and Breast Surgery, Xiaogan Central Hospital affiliated with Wuhan University of Science and Technology, Xiaogan, Hubei, China; Department of Neurosurgery, Xiaogan Central Hospital affiliated with Wuhan University of Science and Technology, Hubei, Xiaogan, China

**Keywords:** breast cancer, touch imprint cytology, diagnosis, sentinel lymph node, meta-analysis

## Abstract

This meta-analysis aimed to evaluate the diagnostic accuracy of touch imprint cytology (TIC) for sentinel lymph node (SLN) metastases of patients with clinical node-negative early breast cancer. The PubMed, Web of Science, Embase, and the Cochrane Library databases were meticulously searched to retrieve literature published from January 2005 to September 2022 by two independent reviewers. The meta-analysis was performed using STATA16.0, Meta-Disc 1.4, and RevMan 5.4.9. According to the inclusion criteria, 4,073 patients from 13 studies were included in this meta-analysis. The pooled sensitivity and specificity of TIC for detecting SLN metastases were 0.77 (95% CI 0.66–0.85) and 0.99 (95% CI 0.97–1.00), respectively. The pooled positive likelihood ratio and negative likelihood ratio were 76.15 (95% CI 29.16–198.84) and 0.23 (95% CI 0.15–0.36), respectively. The pooled diagnostic odds ratio was 326.82 (95% CI 132.76–804.56) and the area under the sROC curve was 0.97 (95% CI 0.95–0.98), respectively. This meta-analysis revealed that TIC with high sensitivity and specificity is a feasibility and accuracy diagnosis technique for intraoperative detection of SLN metastases in breast cancer.

## 1 Introduction

Sentinel lymph node biopsy (SLNB) is a standard procedure for assessing axillary lymph node status in patients with clinically node-negative breast cancer [1,2]. Surgeons usually do not perform axillary lymph node dissection (ALND) in patients with pathologically-negative SLNs, which may avoid many potential and unnecessary complications including lymphedema, paresthesias, limitation of shoulder range of motion, and decline in quality of life [3–5]. However, when intraoperative SLNB demonstrates the detection of SLN metastases, ALND can be completed in a single operation. It can effectively prevent the patients suffering from a second surgical operation, additional pain, and financial burden.

Previous studies have indicated that the intraoperative techniques for evaluation of SLN status contain touch imprint cytology (TIC), frozen section (FS), one-step nucleic acid amplification (OSNA) assay, and immunohistochemistry [6–9]. FS and TIC are the two most commonly used methods for intraoperative evaluation of SLN in the world [10]. In recent years, OSNA and immunohistochemistry technologies have been gradually carried out in some hospitals. In China, FS is the most common method of intraoperative SLN diagnosis in many authoritative medical centers due to its advantage of high sensitivity of detecting macrometastases and micrometastases, and the proportion is surprisingly more than 90%. However, the FS technique is obviously accompanied with many disadvantages such as requirement of more time, tissue loss, and cost. Similarly, these drawbacks also exist in OSNA and immunohistochemistry, which might affect the subsequent histopathological examination [8].

TIC has several limitations such as lack of standardized procedure, minimal node sampling, difficulty in detecting micrometastases and macrometastases from lobular carcinoma, and the requirement of an experienced cytopathologist to interpret the imprints. However, TIC is a rapid, cost-effective, and more tissue conserving technique [11]. Not only that, several studies have demonstrated that TIC has sensitivity equivalent to or better than that of FS [12–15]. Thus, we performed an updated meta-analysis to collect data to evaluate the diagnostic feasibility and accuracy of TIC for detecting SLN metastases in patients with breast cancer.

## 2 Materials and methods

### 2.1 Search strategy

Four databases including PubMed, Embase, Cochrane Library, and Web of Science were systematically searched for articles published from January 2005 to September 2022 by two independent and experienced reviewers. The medical subject heading (Mesh) terms were defined as follows: “breast cancer,” “imprint cytology,” and “sentinel lymph node.” Furthermore, we used combinations of “breast cancer,” “imprint cytology,” and “sentinel lymph node” as free text terms. Meanwhile, the references of selected articles were also carefully screened to identify additional relevant articles.

### 2.2 Inclusion and exclusion criteria

The inclusion criteria were as follows: (1) patients included in the studies were finally diagnosed as breast cancer by postoperative pathology, (2) patients did not receive any neoadjuvant therapy or radiotherapy, (3) the purpose of diagnosis was to detect SLN metastases, (4) all specimens were fresh SLNs, (5) all specimens were evaluated by intraoperative TIC and postoperative histopathology, (6) postoperative hematoxylin–eosin staining technique was used as the reference standard in SLNs assessment, (7) detailed data analysis of all the studies was based on per-patient, and (8) providing sufficient information for calculating false-positive (Fp), false-negative (Fn), true-positive (Tp), and true-negative (Tn) values.

The exclusion criteria were as follows: (1) non-English literature; (2) data analysis of study was based on per-node but not on per-patient; (3) non-clinical research literature including reviews, letters, conference abstracts, basic experiments, case reports, and comments; and (4) published data were insufficient to form a 2 × 2 table.

### 2.3 Data extraction and quality assessment

Data extraction for each included study was performed independently by two reviewers, and then verified for accuracy by another reviewer. Any disagreement was resolved by team consensus through discussion. The following information was extracted: first author, publication year, country, number of patients, number of SLNs, mean age of patients, reference standard method, thickness of slices and interval of serial slices detected by pathological examination, number of Tp, Fp, Fn, and Tn.

Two reviewers independently assessed the quality of included studies using a standardized tool named Quality Assessment of Diagnostic Accuracy Studies (QUADAS-2), which comprises four key domains: patient selection, a reference standard, index testing, and flow and timing. We classified the risk of bias as “low,” “high,” or “unclear.” When the answers are “yes” to all signaling questions for a domain, the risk of bias can be considered low. But if any question is answered “no,” the risk of bias should be judged as high. We used similar criteria to judge the applicability concerns.

### 2.4 Statistical analysis

First, Spearman’s correlation coefficient was used to measure the threshold effect which is an important source of heterogeneity; when *P* > 0.05, there was no threshold effect. Heterogeneity caused by non-threshold effect between the studies was assessed by Cochran *Q*-test and expressed as an “*I*-square” value. *I* ^2^ values <25, 25–50, and >50% indicate mild, moderate, and significant heterogeneity, respectively [16]. If *P* < 0.05 or *I* ^2^ > 50%, which was considered as significant statistical heterogeneity, the combined sensitivity, specificity, negative likelihood ratio (NLR), positive likelihood ratio (PLR), and diagnostic odds ratio (DOR) with the corresponding 95% CIs were calculated by a random-effect model. But if we obtained an insignificant heterogeneity, a fixed effects model was performed [17]. Similarly, Cochran *Q*-test and *I* ^2^ statistics were adopted to evaluate the heterogeneity of the pooled results. We constructed a summary receiver operating characteristic (sROC) curve to evaluate diagnostic accuracy for TIC, the area under the sROC curve (AUC) value is proportional to the diagnostic value. Deek’s funnel plot asymmetry test was performed to assess publication bias.

The statistical analysis was carried out with STATA16.0 (StataCorp, College Station, TX, USA), Meta-Disc 1.4 (Unit of Clinical Biostatistics, Ramo e Cajal Hospital, Madrid, Spain), and RevMan 5.4 (Revman, the Cochrane Collaboration).

## 3 Results

### 3.1 Study selection

**Figure 1:**
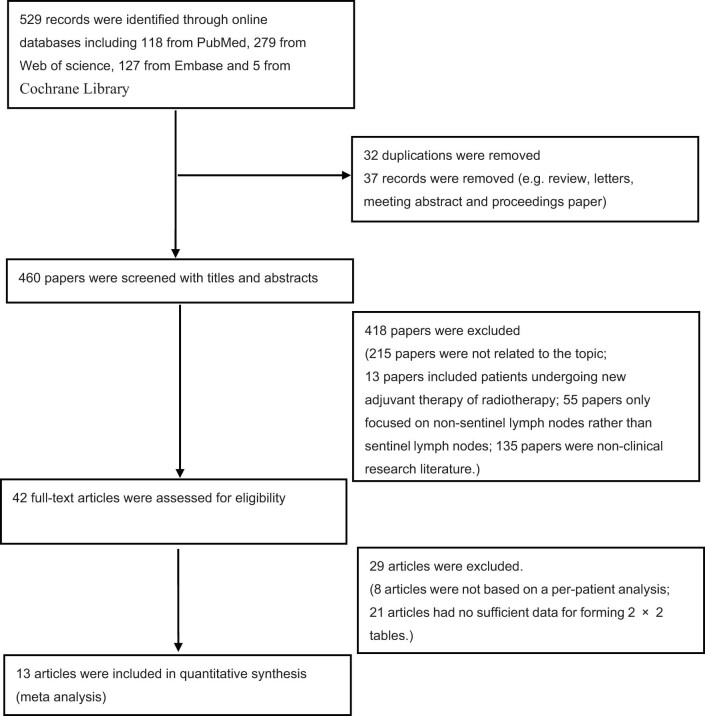
Flow diagram of the study selection process.

As shown in Figure 1, a total of 529 studies from PubMed, Embase, Cochrane Library, and Web of Science databases were retrieved according to the search strategy. We did not find eligible articles after screening the references. The publication dates of these articles ranged from January 2005 to September 2022. Of these, 32 duplicate studies were excluded and 37 studies were removed (e.g., review, letters, meeting abstracts, and proceeding articles). And 447 articles were excluded after screening the titles and abstracts since they did not meet the inclusion criteria: 215 articles were not related to the topic, 13 articles included patients who underwent neoadjuvant therapy or radiotherapy, 55 articles focused on non-sentinel lymph nodes rather than sentinel lymph nodes, 135 articles were non-clinical research literature, data analysis of 8 articles were not based on per-patient, and 21 articles had no sufficient data to form 2 × 2 tables. Finally, 13 articles satisfied the eligibility criteria (Table 1).

**Table 1:** Summary of patient characteristics of included studies

Author	Year	Country	No. of patients	No. of nodes	Mean age	Tp	Fp	Fn	Tn	Thicknesses (μm)	Interval (μm)	Reference method
Cotarelo [18]	2021	Germany	1,072	2,276	61.3	191	0	78	803	2,000	250	H&E
Hashmi [19]	2021	PAK	114	–	53.41 ± 12.46	41	1	8	64	2,000	–	H&E
Pétursson [20]	2018	Sweden	1,227	1,974	60	192	2	88	945	2,000	200	H&E and IHC
Sun [21]	2017	China	79	342	50	12	10	1	56	2,000	200	H&E
Bell [22]	2010	Ireland	102	–	58.8	33	0	8	61	2,000	–	H&E and IHC
Hamidian Jahromi [23]	2009	London	74	146	49.59 ± 13.6	11	1	6	56	–	–	H&E
Upender [24]	2009	India	40	–	49.80 ± 11.19	22	0	2	16	–	–	H&E and IHC
Tamiolakis [25]	2006	Greece	87	128	53.6	24	5	0	58	–	–	H&E and IHC
Clarke [11]	2009	UK	166	239	61.2	29	0	18	119	2,000	–	H&E
Van Eetvelde [26]	2011	Belgium	45	83	60	13	1	9	22	2,000	300	H&E and IHC
Motomura [27]	2008	Japan	631	–	54	110	17	20	484	2,000	200	H&E and IHC
Seenu [28]	2021	India	81	176	49.88	10	1	6	64	2,000	–	H&E
Lorand [29]	2010	France	355	–	56.9	32	2	56	265	2,000	250	H&E and IHC

### 3.2 Study characteristics

The 13 eligible articles (Cotarelo et al., 2021; Hashmi et al., 2021; Pétursson et al., 2018; Sun et al., 2017; Bell et al., 2010; Hamidian Jahromi et al., 2009; Upender et al., 2009; Tamiolakis et al., 2006; Motomura et al., 2008; Seenu et al., 2021; Clarke et al., 2009; Lorand et al., 2010; Van Eetvelde et al., 2011) containing a total of 4,073 patients were included in this meta-analysis [11,18–29]. There were two approaches of pathological examinations in different studies: six studies adopted H&E staining and seven studies combined detection of H&E and IHC.

### 3.3 Quality assessment

**Figure 2:**
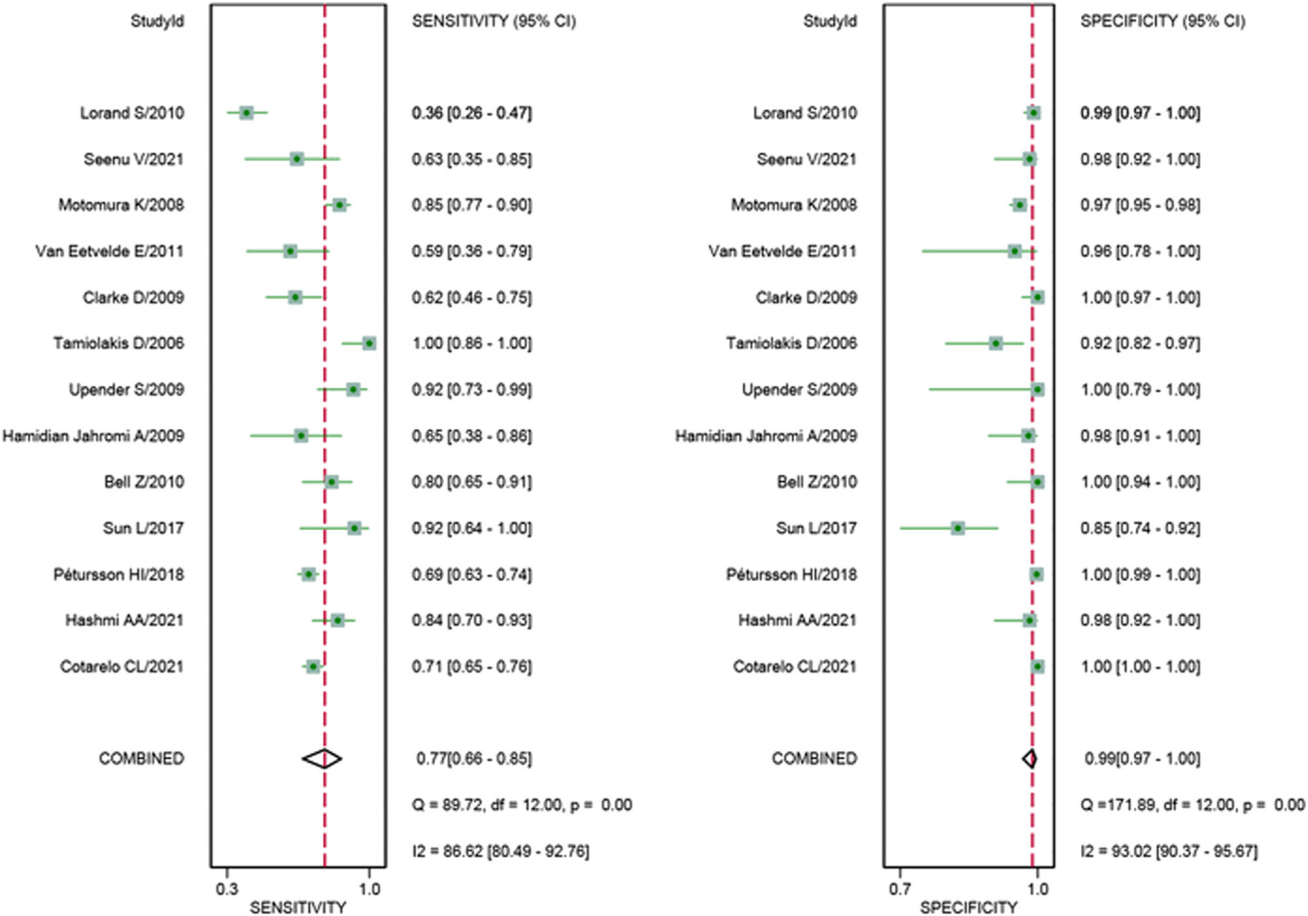
Forest plots of pooled sensitivity and specificity.

The QUADAS-2 was used to assess the quality of each study. Each question was evaluated separately by two independent reviewers, and the disputed issues were evaluated again by a third party to reach a consensus. The results are shown in Figure 2, which indicate that the risk of bias was low and the quality was moderate to high.

### 3.4 Diagnostic performance

**Figure 3:**
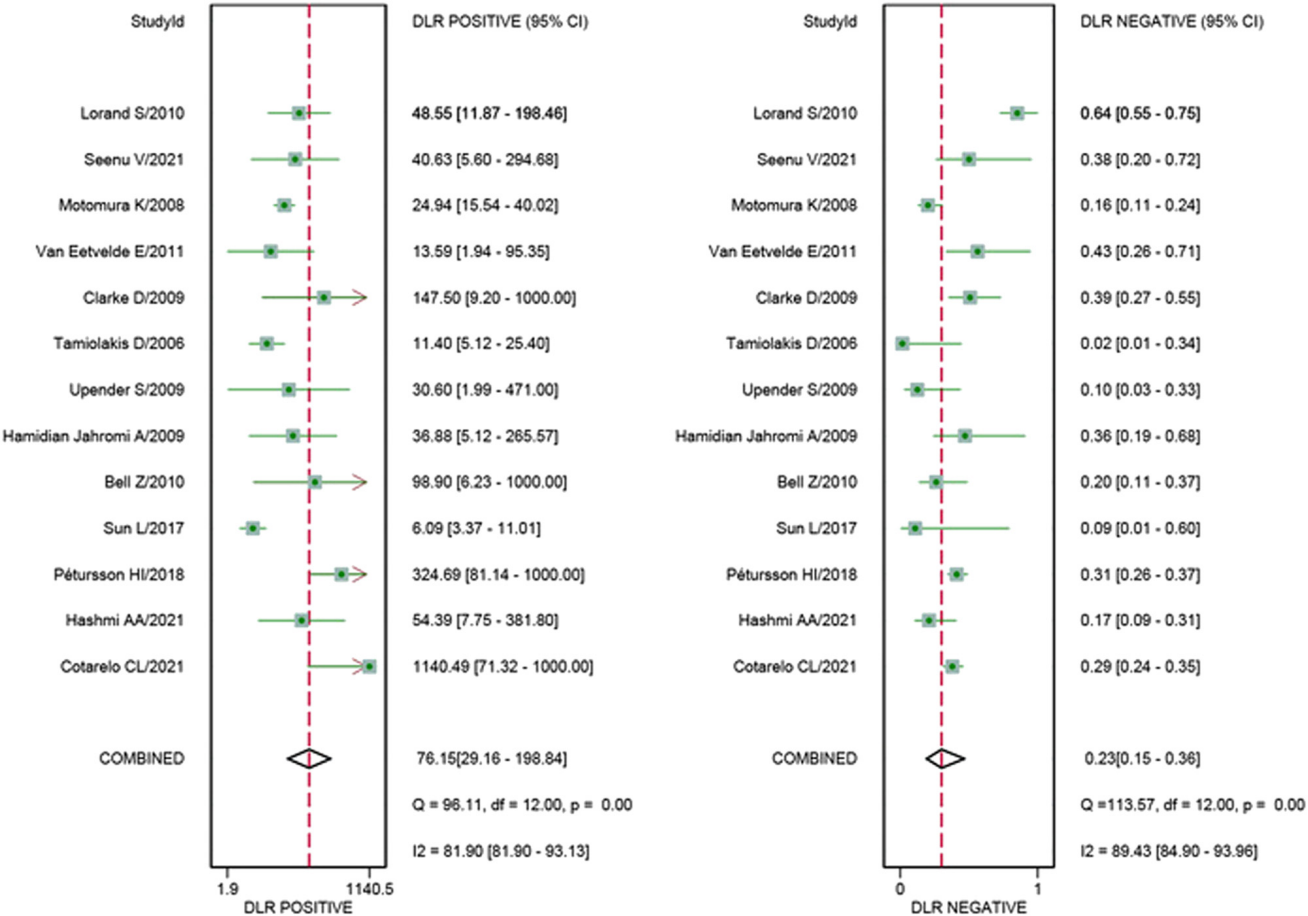
Forest plots of pooled PLR and NLR.

**Figure 4:**
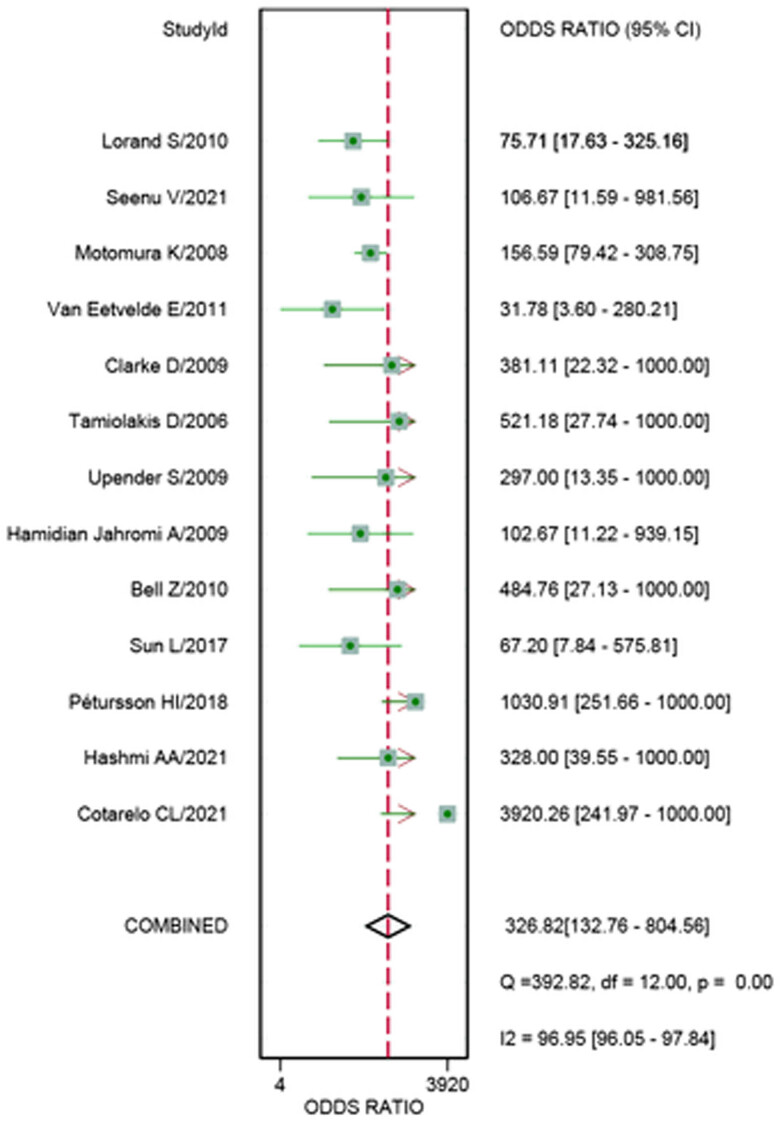
Forest plots of pooled DOR.

**Figure 5:**
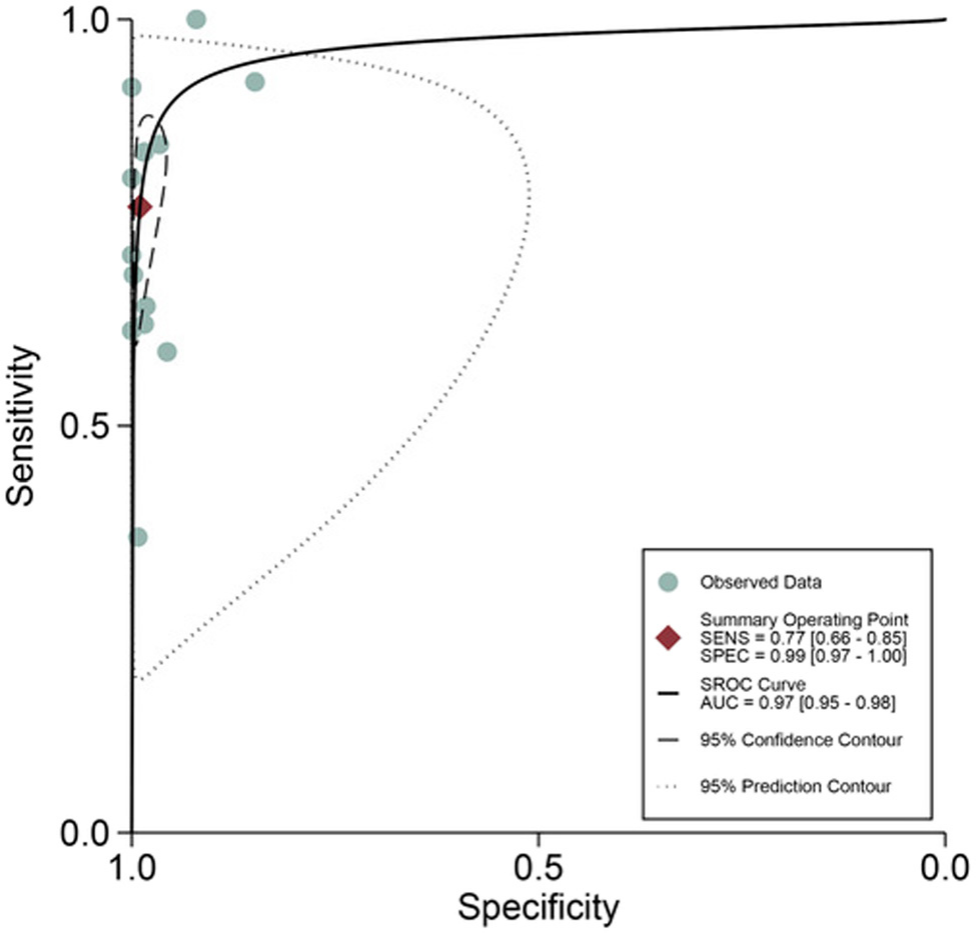
ROC curve for the diagnostic accuracy of TIC in detecting SLN metastasis in breast cancer.

In the threshold analysis, Spearman’s correlation coefficient was 0.45 and *P*-value was 0.12, which indicated that there was no evidence of a threshold effect. We adopted a random effect model since the significant heterogeneity caused by non-threshold effects to perform the follow-up meta-analysis. According to Figure 2, we figured out that the values of *I* ^2^ for sensitivity and specificity were 86.62 and 93.02%, respectively. The pooled sensitivity and specificity (Figure 2) of TIC for detecting SLN metastases were 0.77 (95% CI 0.66–0.85) and 0.99 (95% CI 0.97–1.00), respectively. The pooled PLR and NLR (Figure 3) were 76.15 (95% CI 29.16–198.84) and 0.23 (95% CI 0.15–0.36), respectively. The pooled DOR (Figure 4) was 326.82 (95% CI 132.76–804.56) and AUC (Figure 5) was 0.97 (95% CI 0.95–0.98), respectively.

### 3.5 Publication bias

**Figure 6:**
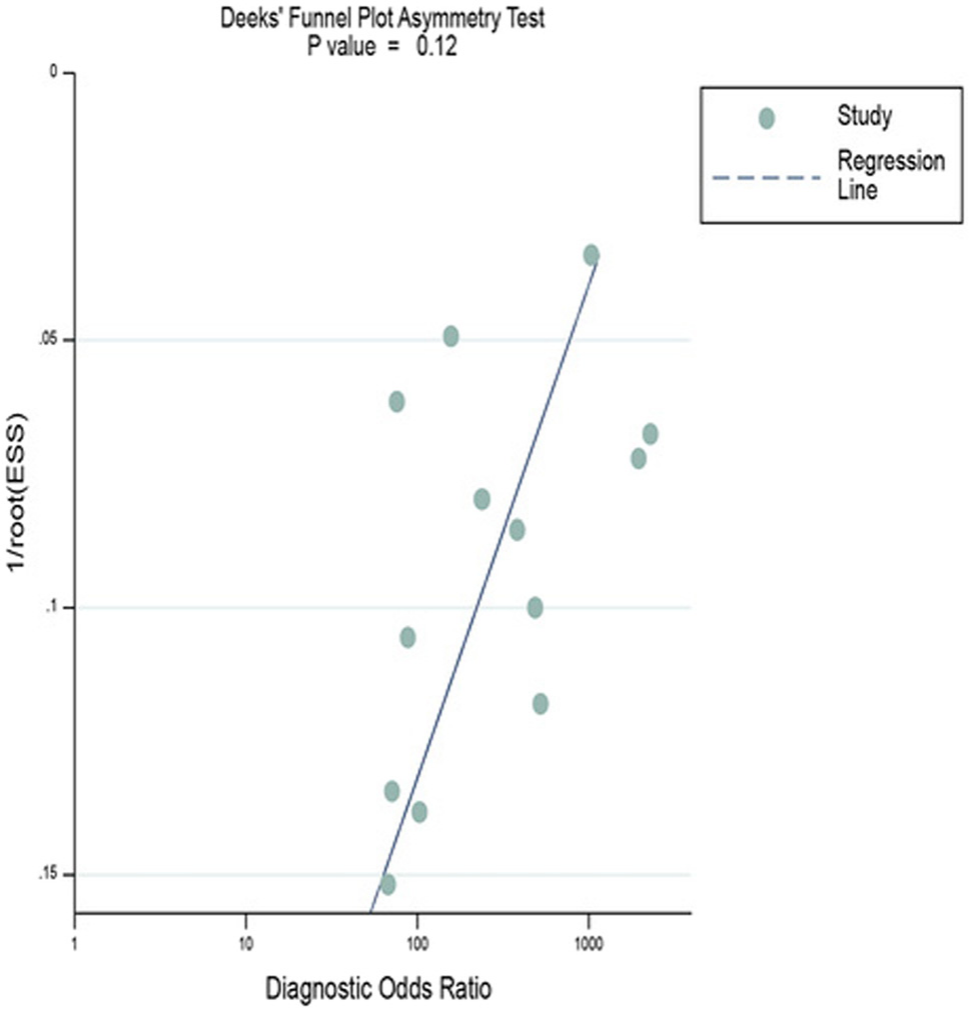
Deek’s funnel chart asymmetry test of publication bias.

The evaluation of the publication bias among the included studies was carried out by performing Deek’s funnel chart asymmetry test using STATA software. There was no evidence of significant publication bias since the shape of Deek’s funnel chart was symmetrical and *P* value was 0.12 (Figure 6).

## 4 Discussion

There is no doubt that axillary lymph node status is a very important prognostic factor of invasive breast cancer. SLNB has become a routine method to predict lymph node status [30]. Since it not only enables patients with SLN metastases to complete ALND in one operation, but also enables patients with negative SLN to avoid suffering from long-term complications, such as upper limb lymph node edema, pain, numbness, etc. [3–5].

It is known that more than 90% of breast centers in China only use FS as a technique for intraoperative evaluation of SLN metastases in breast cancer. However, given the disadvantages of FS such as expensive, loss of tissue, time-consuming, and requirement of experienced and trained technologists and pathologists [8], some authoritative hospitals at home and abroad focused on TIC in view of its main advantages such as simple, rapid, cost-effective, and leaving more tissues for final postoperative pathological analyses [11].

Although some studies have reported the specificity and sensitivity of TIC for the SLN intraoperative diagnosis in breast cancer, the sample sizes of these studies are relatively moderate and the results vary to some extent. Therefore, we conducted the comprehensive meta-analysis to comprehensively analyze the accuracy of TIC in the intraoperative diagnosis of SLN in breast cancer, in the hope that both doctors and patients could benefit from it. In this study, we meta-analyzed 13 studies based on per-patient to evaluate the diagnosis value of TIC. We figured out that the pooled sensitivity, specificity, PLR, NLR, DOR, and AUC of TIC were 0.77 (95% CI 0.66–0.85), 0.99 (95% CI 0.97–1.00), 76.15 (95% CI 29.16–198.84), 0.23 (95% CI 0.15–0.36), 326.82 (95% CI 132.76–804.56), and 0.97 (95% CI 0.95–0.98), respectively. As reported by Kim et al., higher PLR values forecast a stronger association between a test result and a disease, but lower NLR values indicate a stronger association between a test result and the absence of a disease. Thus, given the PLR and NLR values obtained for TIC, this technique is reliable for the intraoperative evaluation of SLN metastases in breast cancer. Furthermore, the sROC value was 0.97, which proved a high degree of overall diagnostic accuracy [31].

Numerous studies have shown that the sensitivity of TIC for the diagnosis of macrometastasis is comparable to FS. However, TIC has a low sensitivity in the diagnosis of micrometastasis. Studies indicated that pathologists with proper technique and experience are more likely to detect micrometastasis. Moreover, even if the micrometastasis is detected in postoperative pathology, there is no necessity to perform ALND. This view has been recognized by the expert consensus [32]. Previous studies have shown that TIC is more accurate than FS in diagnosis of negative SLNs, which means that TIC has a better negative predictive value than FS [21]. The SLN metastasis in invasive lobular carcinoma is difficult to be diagnosed because of the different subtypes of lobular carcinoma with features of diverse cytological morphology. For instance, rosette-like pattern is usually detected in alveolar type; large cell is more common in the polymorphic type. Unfortunately, these subtypes are difficult to be identified except for some very experienced pathologists by TIC [33]. Although the sensitivity of TIC technique in detecting micrometastasis and metastatic sentinel nodes in invasive lobular carcinoma is not satisfactory, it has many obvious advantages such as simplicity, leaving more tissue for postoperative histopathological examination, and saving operative time and cost for some patients as compensation. More encouragingly, it has been reported that the sensitivity of TIC is increasing over time [34].

Although we have conducted a comprehensive and rigorous analysis, there are still several limitations in our present study. First, we only adopted the literature published in English and excluded potential studies published in other languages. Second, our meta-analysis only incorporated published research. However, studies with meaningful positive results are much more likely to be published than those with negative results, which can lead to potential publication bias. Third, there were several factors contributing to clinical and methodological heterogeneity in our analysis, such as the populations of the study samples, proportions of patients with different age, tumor size, molecular subtype and histological grade, and reagent manufacturer selected. Unfortunately, when we tried to perform subgroup analysis, we found some data deficiencies.

## 5 Conclusions

Intraoperative evaluation of SLN in patients with clinically node-negative breast carcinoma by TIC is proved to be a reliable and feasible technique. TIC and FS showed similar low sensitivity in the diagnosis of micrometastasis. However, TIC retains more tissue for subsequent histological evaluation as compensation. As a result, we believe that the TIC technique for diagnosis of SLN metastasis in breast cancer deserves to be widely applied in clinical practice.

## Abbreviations

ALND: axillary lymph node dissection
AUC: area under the sROC curve
DOR: diagnostic odds ratio
FS: frozen section
NLR: negative likelihood ratio
OSNA: one-step nucleic acid amplification
PLR: positive likelihood ratio
QUADAS: Quality Assessment of Diagnostic Accuracy Studies
SLNB: sentinel lymph node biopsy
SROC: Summary receiver operating characteristic
TIC: touch imprint cytology
